# Effects of Lipopolysaccharide and Deoxynivalenol on the Survival, Antioxidant and Immune Response, and Histopathology of Crayfish (*Procambarus clarkii*)

**DOI:** 10.3390/toxins15080479

**Published:** 2023-07-28

**Authors:** Zhengrong Wen, Xiaoli Xu, Dan Xiang, Junfeng Xu, Qiufeng Yang, Xiaofu Wang, Jiashou Liu, Mingzhong Luo, Wei Wei

**Affiliations:** 1 Engineering Research Centre of Ecology and Agricultural Use of Wetland, Ministry of Education, Hubei Key Laboratory of Waterlogging Disaster and Agricultural Use of Wetland, College of Animal Science, Yangtze University, Jingzhou 434025, China; wzr97329@163.com (Z.W.); xiangdan409@163.com (D.X.); yangqiufen88@126.com (Q.Y.); 2 State Key Laboratory for Managing Biotic and Chemical Threats to the Quality and Safety of Agro-Products, Key Laboratory of Traceability for Agricultural Genetically Modified Organisms, Ministry of Agriculture and Rural Affairs, Zhejiang Academy of Agricultural Sciences, Hangzhou 310021, China; xuxiaoli@zju.edu.cn (X.X.); njjfxu@163.com (J.X.); yywxf1981@163.com (X.W.); 3 State Key Laboratory of Freshwater Ecology and Biotechnology, Institute of Hydrobiology, Chinese Academy of Sciences, Wuhan 430072, China; jsliu@ihb.ac.cn

**Keywords:** crayfish, deoxynivalenol, lipopolysaccharide, survival, antioxidant and immune response, histopathological changes

## Abstract

Bacterial lipopolysaccharide (LPS) in the aquatic environment has been reported to cause diseases in red swamp crayfish (*Procambarus clarkii*). In addition, deoxynivalenol (DON) is one of the primary mycotoxins found in aquaculture. However, the potential synergistic toxic effects of LPS and DON on crayfish are yet to be fully elucidated. In this study, crayfish were exposed to LPS (1 mg kg^−1^), DON (3 mg kg^−1^), and their combination (1 mg kg^−1^ LPS + 3 mg kg^−1^ DON, L+D) for a duration of six days. Co-exposure to LPS and DON exhibited the lowest survival rate compared to the control or individual treatments with LPS or DON alone. In the initial stage of the experiment, the combined treatment of LPS and DON showed a more pronounced up-regulation of antioxidant and immune-related enzymes in the sera compared to the other treatment groups, with a fold change ranging from 1.3 to 15. In addition, the (L+D) treatment group showed a down-regulation of immune-related genes, as well as Toll pathway-related genes in the hepatopancreas compared to LPS or DON. Moreover, the (L+D) treatment group demonstrated a 100% incidence of histopathological changes in the hepatopancreas, which were significantly more severe compared to the other three groups. In conclusion, our study provides physiological and histopathological evidence that the co-exposure to LPS and DON exerted synergistic toxic effects on crayfish. The observed effects could potentially hinder the development of the crayfish aquaculture industry in China.

## 1. Introduction

The red swamp crayfish (*Procambarus clarkii*) is a freshwater crayfish species that has adapted to a wide range of aquatic environments [1]. In China, crayfish is currently one of the most crucial freshwater aquaculture species [2]. However, the high abundance of bacteria, viruses, and parasites found in aquatic environments poses a severe threat to crayfish health and production [3]. Gram-negative bacteria can trigger a systemic immune response, leading to sepsis, pneumonia, and gastrointestinal disease [4], partly due tolipopolysaccharide (LPS), a heat-stable endotoxin and cell wall component of Gram-negative bacteria [5]. LPS has been reported to activate the crayfish prophenoloxidase activation system (proPO-activating system) and induce crayfish melanization [6,7].

Deoxynivalenol (DON), a trichothecene mycotoxin, is the most prevalent mycotoxin found in aquafeeds, with a maximal DON concentration level of approximately 1 mg kg^−1^ [8]. Due to its high prevalence and widespread occurrence in livestock feeds, DON is considered a predominant environmental risk to animal productivity [9,10]. The European Commission has established a general recommendation of guidance values of 5 mg kg^−1^ DON in feedstuff (2006/576/EC) (European Commission, 2006) [11]. Consequently, this mycotoxin can be transferred to aquatic environments. Previous studies have reported that some species of fish, such as rainbow trout, are reported to be highly sensitive to feed-borne DON [12], as well as DON can stimulate the immune response of crustaceans [13]. A low concentration of DON in the diet of white shrimp (*Litopenaeus vannamei*) can damage the intestinal mucosal structure, reduce growth rate and survival rate, and impact muscle quality [14]. Previous studies have reported that DON in aquatic environments can reduce metabolic activity and cell viability, as well as reactive oxygen species (ROS) production in established permanent fish cell lines derived from rainbow trout [15]. Additionally, DON can induce apoptosis and necrosis of neutrophils in common carp [16]. Recently, a study reported a new mechanism of DON toxicity, revealing that ferroptosis is involved in DON-induced intestinal damage in pigs [17]. However, the toxicity of DON on crayfish has not yet been investigated.

Despite the fact that aquatic environments often contain a mixture of Gram-negative bacteria, such as pathogenic *Vibrio* species, and feed-borne mycotoxins, few ecotoxicological evaluations of the combined toxicity of bacterial LPS and trichothecene DON have been conducted on aquatic animals. Current studies on the combined toxicity of LPS and DON have demonstrated that LPS priming sensitizedmice to DON-induced proinflammatory cytokine induction and apoptosis [18]. LPS challenge induced an up-regulation of the proinflammatory response in the duodenum and enhanced the mucosal permeability in the jejunum of broiler chickens [19]. However, the LPS challenge did not result in any significant effect on the transport of DON across porcine jejunal mucosa [20]. Therefore, further studies are required to investigate the potentially toxic effects of DON as well as the synergistic toxic effects of LPS and DON on crayfish.

In this study, we aimed to assess the potentially toxic effects of bacterial LPS, a common feed-borne mycotoxin DON, and their combination on the survival rate, activities of antioxidant and immune-related enzymes, expression levels of immune-related genes, and histopathology of crayfish.

## 2. Results

### 2.1. Survival Rate

**Figure 1:**
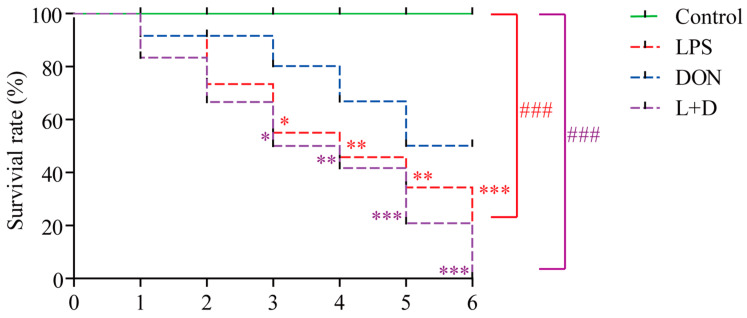
The survival rate of crayfish exposure to the LPS, DON, and L+D treatments. Crayfish were treated with LPS (1 mg kg^−1^), DON (3 mg kg^−1^), or L+D (1 mg kg^−1^ LPS + 3 mg kg^−1^ DON) via injection. The death of crayfish in each group was recorded daily. Data are presented as mean ± SEM (*n* = 12). ### *p* < 0.001 (two-way ANOVA test), * *p* < 0.05, ** *p* < 0.01, *** *p* < 0.001 versus control (Tukey’s multiple comparison test).

At the end of the experiment, there were no deaths in the Control group. The survival rates of crayfish were 66.67%, 33.33%, and 16.67% in the DON, LPS, and (L+D) treatment groups, respectively. Compared to the control group, crayfish in the DON treatment group did not show a significant difference. However, crayfish in the LPS and (L+D) groups had significantly lower survival rates throughout the experiment. There was no significant difference between the groups with LPS and (L+D) treatments (Figure 1).

### 2.2. Activities of Enzyme and Expressions of Enzyme-Related Genes

**Figure 2:**
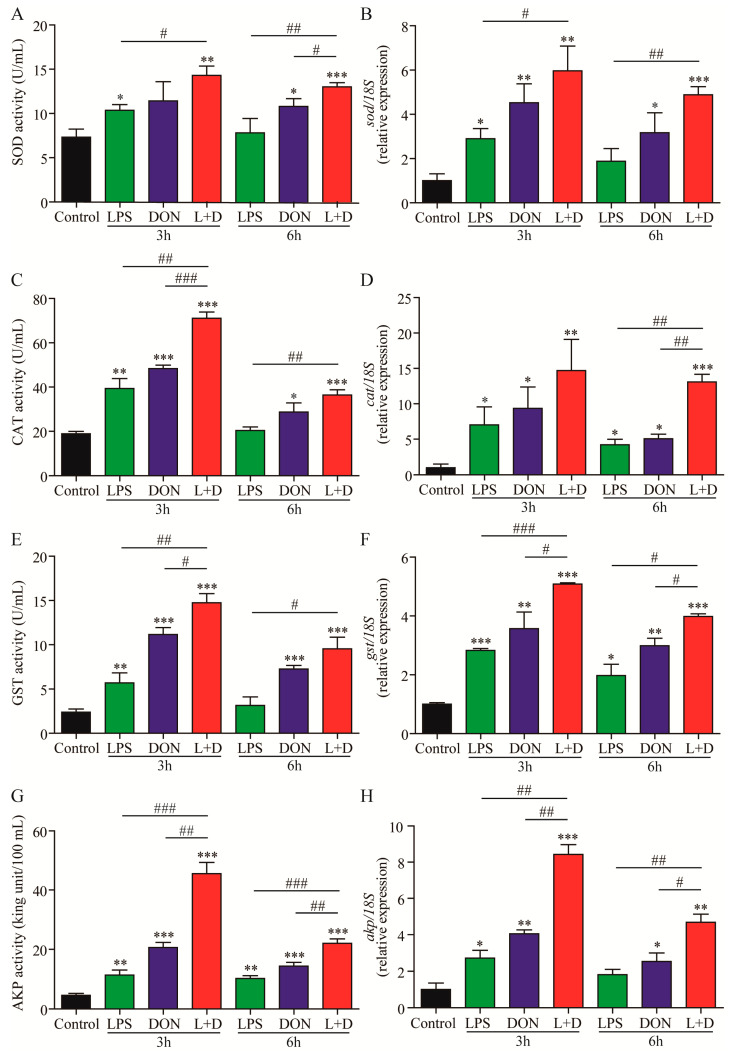
Antioxidant and immune-related enzymes of crayfish exposure to the LPS, DON, and L+D treatments. The serum and hepatopancreas samples of crayfish were collected at 3 and 6 h, and the enzyme activities (SOD (**A**), CAT (**C**), GST (**E**), and AKP (**G**)) in sera and gene expressions (*sod* (**B**), *cat* (**D**), *gst* (**F**), and *akp* (**H**)) in hepatopancreas were measured using ELISA and RT-qPCR analysis, respectively. Data are presented as mean ± SEM (*n* = 4). * *p* < 0.05, ** *p* < 0.01, *** *p* < 0.001 versus control; # *p* < 0.05, ## *p* < 0.01, ### *p* < 0.001 (Tukey’s multiple comparison test).

The activities of antioxidant enzymes (superoxide dismutase (SOD), catalase (CAT), and glutathione S-transferase (GST)) and immune-related enzymes (alkaline phosphatase, AKP) in sera were measured using ELISA, while the expression levels of enzyme-related genes in the hepatopancreas of the four groups were measured using RT-qPCR analysis. Compared to the Control group, the DON treatment group showed significantly higher activities of SOD, CAT, GST, and AKP at both 3 and 6 h. Additionally, the enzymatic activities in the (L+D) treatment group were significantly higher than those in the DON or LPS treatment groups (Figure 2A,C,E,G). Correspondingly, the expression levels of *sod*, *cat*, *gst,* and *akp* in the (L+D) treatment group were also up-regulated and significantly higher than those in the LPS or DON treatment groups at different time points (Figure 2B,D,F,H), consistent with the results of enzymatic assays. Our findings suggest that acute injection of LPS and DON synergistically stimulate oxidative stress and immune response in crayfish.

### 2.3. Expressions of Immune-Related Genes

**Figure 3:**
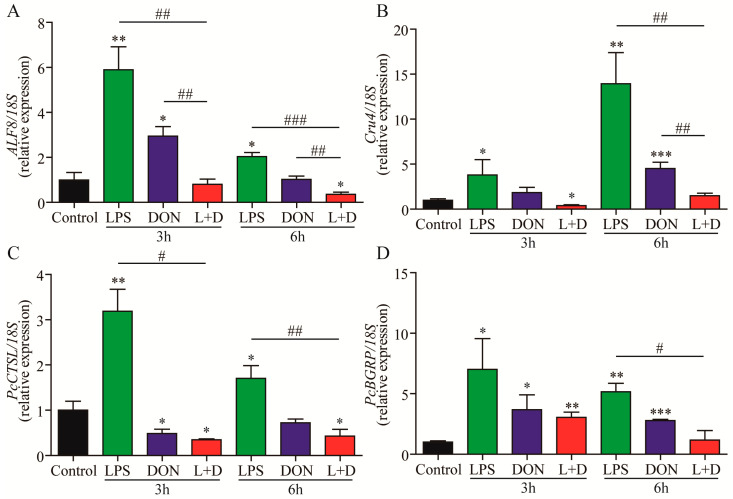
The expressions of immune-related genes in the hepatopancreas of crayfish exposure to the LPS, DON, and L+D treatments. The hepatopancreas samples of crayfish were collected at 3 and 6 h, and the gene expressions (*ALF8* (**A**), *Cru4* (**B**), *PcCTSL* (**C**), and *PcBGRP* (**D**)) in hepatopancreas were measured using RT-qPCR analysis. Data are presented as mean ± SEM (*n* = 4). * *p* < 0.05, ** *p* < 0.01, *** *p* < 0.001 versus control; # *p* < 0.05, ## *p* < 0.01, ### *p* < 0.001 (Tukey’s multiple comparison test).

The expression levels of innate immune-related genes, including anti-lipopolysaccharide factors 8 (*ALF8*), crustin 4 (*Cru4*), cathepsin-L (*PcCTSL*), and β-1, 3-glucosidase related protein (*PcBGRP*), were quantified in the hepatopancreas of the four groups using RT-qPCR analysis. At both 3 and 6 h, the expressions of *ALF8*, *Cru4*, *PcCTSL*, and *PcBGRP* in the LPS treatment group were significantly up-regulated compared to the Control group (Figure 3). In the DON treatment group, the expressions of *ALF8*, *Cru4*, and *PcBGRP* were significantly up-regulated (Figure 3A,B,D), but the expression of *PcCTSL* was slightly down-regulated at 3 h compared to the Control group (Figure 3C). In the (L+D) treatment group, the expressions of *ALF8* and *Cru4* were significantly down-regulated compared to the LPS or DON treatment group (Figure 3A,B), and the expressions of *PcCTSL* and *PcBGRP* were significantly down-regulated compared to the LPS treatment group (Figure 3C,D). However, there were no significant differences in the expressions of *PcCTSL* and *PcBGRP* between the DON and (L+D) treatment groups (Figure 3C,D). Taken together, the combined treatment of LPS and DON synergistically down-regulated the expressions of immune-related genes, thereby regulating the crayfish’s innate immune system.

### 2.4. Expressions of Toll Pathway-Related Genes

**Figure 4:**
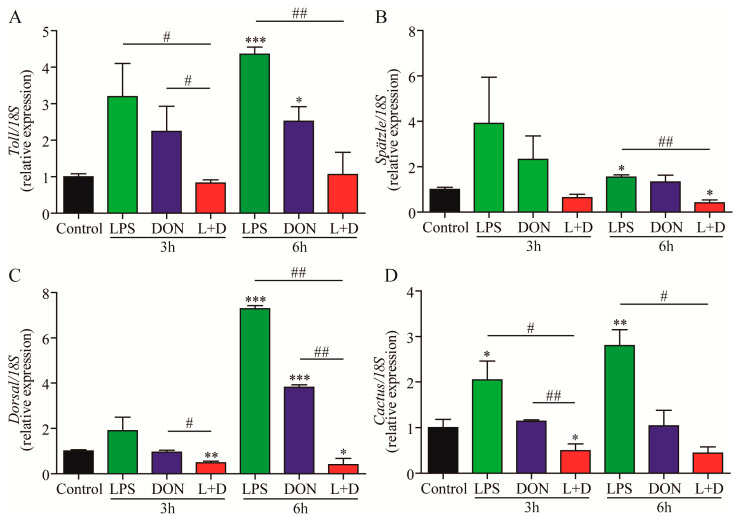
The expression of Toll pathway-related genes in the hepatopancreas of crayfish exposure to the LPS, DON, and L+D treatments. The hepatopancreas samples of crayfish were collected at 3 and 6 h, and the gene expressions (*Toll* (**A**), *Spätzle* (**B**), *Dorsal* (**C**), and *Cactus* (**D**)) in hepatopancreas were measured using RT-qPCR analysis. Data are presented as mean ± SEM (*n* = 4). * *p* < 0.05, ** *p* < 0.01, *** *p* < 0.001 versus control; # *p* < 0.05, ## *p* < 0.01 (Tukey’s multiple comparison test).

The expression levels of Toll pathway-related genes, including *Toll*, *Spätzle*, *Dorsal*, and *Cactus*, were quantified in the hepatopancreas of the four groups using RT-qPCR analysis. At 6 h, the expressions of *Toll*, *Spätzle*, *Dorsal*, and *Cactus* in the LPS treatment group were significantly up-regulated compared to the Control group (Figure 4). In the DON treatment group, the expressions of *Toll* and *Dorsal* were significantly up-regulated compared to the control group (Figure 4A,C). In the (L+D) treatment group, the expressions of *Toll*, *Dorsal*, and *Cactus* were significantly down-regulated compared to the LPS or DON treatment group (Figure 4A,C,D), and the expression of *Spätzle* was significantly down-regulated compared to the LPS treatment group (Figure 4B). However, there was no significant difference in the expression of *Spätzle* between the DON and (L+D) treatment groups (Figure 4B). Our study found that the mixtures of LPS and DON had a synergistic effect on down-regulated expressions of Toll pathway-related genes. 

### 2.5. Histological Changes in Hepatopancreas

**Figure 5:**
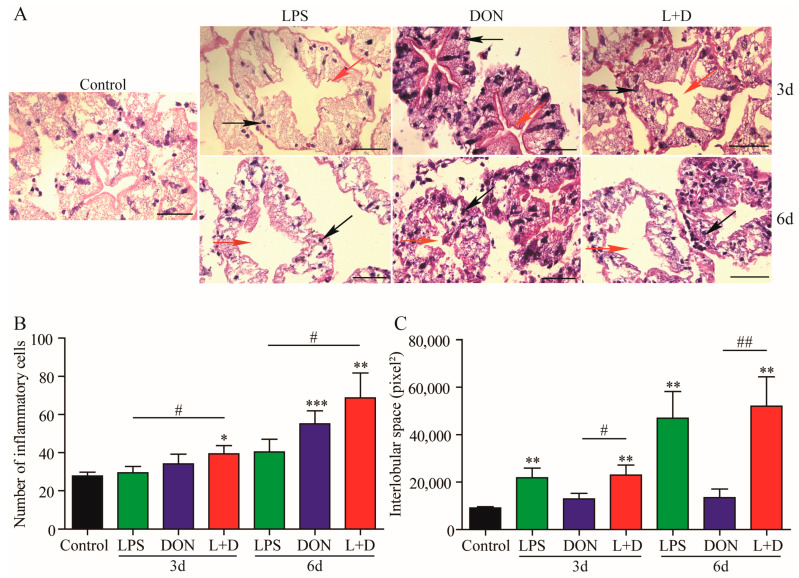
Histopathological changes in the hepatopancreas of crayfish exposure to the LPS, DON, and L+D treatments. The hepatopancreas samples of crayfish were collected on Days 3 and 6. (**A**) H&E staining, bar: 50 μm, black arrows point to inflammatory cells, and red arrows point to hepatic interlobular spaces. The inflammatory cells (**B**) of crayfish were counted, and the area of hepatic interlobular space (**C**) was recorded by Image-pro Plus 6.0 software; the area of hepatic interlobular space was 100 pixels. Data are presented as mean ± SEM (*n* = 10). * *p* < 0.05, ** *p* < 0.01, *** *p* < 0.001 versus control; # *p* < 0.05, ## *p* < 0.01 (Tukey’s multiple comparison test).

The histological changes in the hepatopancreas of crayfish with different treatments were examined using H&E staining. Compared to the Control group, there was a significant increase in inflammatory cells in the (L+D) treatment group on Day 3, and the number of inflammatory cells was greatly increased in the DON and the (L+D) treatment groups on Day 6 (Figure 5A,B). Although the number of inflammatory cells in the (L+D) group was higher than that in the DON group, there was no difference between the DON and the (L+D) groups (Figure 5B). With regard to histological alterations, the hepatic lobular space was significantly enlarged in the (L+D) treatment group compared to that in the Control and DON treatment groups on days 3 and 6, but there was no difference between the LPS and the (L+D) groups (Figure 5A,C). Overall, the mixture treatment with LPS and DON exacerbated histological changes in the hepatopancreas of crayfish.

## 3. Discussion

DON is highly water-soluble and relatively stable in aquatic environments, making it a potential source of water environmental pollutants in crayfish culture. Despite this, the combined toxicity of LPS and DON has only been studied in terrestrial animal models, such as mice, chickens, and pigs [18,19,20]. In this study, we found that both LPS and the combination treatment of LPS and DON significantly reduced the survival rate of crayfish, while there was no significant effect on the mortality of crayfish treated individually with DON compared to the control group. Consistently, previous studies have reported a 20% mortality rate of white shrimp (*Litopenaeus schmitti*) within 24 h of LPS injection [21], as well as an increased mortality rate of mice within 40 h of combined treatment of LPS and DON. However, LPS treatment did not affect the survival of mice [22]. This difference may be due to the different modes of LPS administration as well as the different species used in the studies. To the best of our knowledge, this is the first report documenting the combined toxicity of LPS and DON on aquatic animals. Therefore, both the DON and LPS released from dividing and dead bacteria may pose a threat to the health of crayfish. 

Although DON did not have a significant effect on the mortality of crayfish, it has been reported to alter the levels of primary antioxidant enzymes, such as SOD, CAT, and GST, in various species or cell lines [23]. In this study, the levels of SOD, CAT, and GST in sera, as well as the transcription of enzyme-related genes in the hepatopancreas, which is an important immune and metabolic organ in crayfish [24], were significantly increased in the DON treatment group. Our results were consistent with those of a study on human hepatocellular carcinoma HepG2 cells treated with DON [25]. Additionally, the combined treatment of LPS and DON significantly induced the highest activities of SOD, CAT, GST, and AKP among the different treatment groups, as well as relative gene expression, particularly for the unconventional immune protein AKP. This leads us to hypothesize that the combined treatment of DON and LPS may have an impact on the immune response of crayfish. The intestinal microflora performs many crucial functions essential for the host’s health [26]. Lucke et al. found that the combined treatment of LPS and DON could change the composition of microflora; volatile fatty acids in the chicken cecum could reduce the diversity of microflora [27]. Similarly, in our study, treatment with (L+D) decreased the intestinal microbial diversity and changed the composition of the intestinal flora. Interestingly, sudden changes in the abundance of inherent microflora in the intestinal tract have been reported to induce intestinal and systemic immune diseases [28,29]. Furthermore, treatment with (L+D) can significantly decrease the abundance of beneficial bacteria *Candidatus_Bacilloplasma* [30] and inherent microflora *Bacteroidetes* and *Tenericutes*, while the number of disease-related bacteria such as *Proteobacteriacan*, *Citrobacter*, *Vibrio*, and *Shewanella* is significantly increased [31,32]. This shows that the combined treatment of LPS and DON may change the diversity of the intestinal flora of crayfish by changing the ratio of harmful and beneficial bacteria in the intestinal flora of crayfish and disrupting the internal balance of the intestine. In a nutshell, up-regulation of the activities of immune enzymes and down-regulation of the expression of immune genes in the (L+D) treatment group resulted in hepatopancreatic injury and, ultimately, death of crayfish; LPS and DON exert a synergistic toxic effect on crayfish. However, the specific mechanism underlying the effects of LPS and DON on the immune cells of aquatic animals warrants further investigation. 

Innate immunity is crucial for crayfish, which lack an adaptive immune system [33] and is primarily activated through the Toll pathway and immune-deficiency (IMD) pathway [34]. It is widely recognized that ALF8, Cru4, PcCTSL, and PcBGRP are important immune effector molecules, while Spätzle, Dorsal, and Cactus are related to the Toll signaling pathway in crustaceans. Our results correspond to previous studies which reported that LPS challenge up-regulated the expression levels of immune effector molecules, such as PcCTSL and PcBGRP, in the hepatopancreas of crayfish [35,36]. In a previous study, the intestinal permeability of the duodenum in broiler chickens treated with LPS and DON was altered, and the expressions of the Toll signaling pathway and inflammation-related genes were up-regulated [19]. However, in our study, the transcription of *Toll*, *Spätzle*, *Dorsal*, and *Cactus* related to the Toll signaling pathway, as well as the expressions of inflammation-related genes (*ALF8,Cru4*, *PcCTSL*, and *PcBGRP*), were down-regulated in the (L+D) treatment group. The down-regulation of immune-related gene expression in the hepatopancreas of crayfish may be attributed to histological alterations and the impairment of the innate immune system of crayfish resulting from the combined treatment of LPS and DON [37].

China is the world’s largest crayfishproducer, contributing to over 90% of the global annual output [38]. In 2022, the total economic output of crayfish aquaculture reached 458 billion yuan, solidifying the position of crayfish as the most significant freshwater aquaculture species in the country. Based on our findings, the treatment with LPS and DON showed a significant decrease in crayfish survival. If such situations occur in crayfish aquaculture, it could lead to economic losses amounting to billions of yuan and pose a severe threat to the industry. Given the demonstrated potential of bacterial toxins, such as LPS and mycotoxins, such as DON, to adversely affect crayfish culture, implementing effective measures to control the contamination of DON or LPS becomes crucial for safeguarding the crayfish industry. We recommend implementing the following strategies in crayfish aquaculture to address the issue: Firstly, it is essential to conduct regular mycotoxin detection in the feed before feeding it to crayfish. This step ensures that the feed is free from mycotoxin contamination, minimizing the risk of negative impacts on crayfish health. Secondly, maintaining strict control over the water environment is crucial. This control helps to prevent the proliferation of pathogenic bacteria and subsequently reduces their bioproduction. By adopting these strategies, the contamination of mycotoxins such as DON, and bacterial toxins such as LPS, can be minimized, thereby protecting the crayfish industry in China.

## 4. Conclusions

Physiological and pathological analyses were conducted to evaluate the acute combined toxicity of LPS and DON on crayfish. The results showed that a mixture of LPS and DON had synergistic toxic effects on crayfish. Specifically, co-exposure to LPS and DON significantly up-regulated the antioxidant enzymes and down-regulated the immune-related and Toll pathway-related genes compared to those with exposure to LPS or DON alone. Moreover, the (L+D) treatment caused severe tissue injury in the hepatopancreas, resulting in increased mortality of crayfish, with toxicity proportional to treatment time. However, to fully understand the cytotoxic mechanism of LPS and DON on crayfish, further investigation using molecular biology and immunological methods is warranted.

## 5. Materials and Methods

### 5.1. Animals and Chemicals

The crayfish used in this study were obtained from a crayfish farm in Jingzhou, China, and had an average weight of 15 ± 1.2 g (mean ± SEM). The crayfish were fed commercial aquafeeds once a day and were cultured in an aquarium (120 cm × 30 cm × 65 cm, L:W:H) at 24 ± 2 °C under a photoperiod of 14: 10 h (light:dark cycle) for seven days. LPS from *E. coli* O55:B5 (≥500,000 EU/mg) was purchased from Biosharp (Guangzhou, China), and DON with a purity of >99.0% was purchased from Pribolab (Qingdao, China). The LPS and DON working solutions were prepared by diluting them with 0.9% sterile saline solution.

### 5.2. Experimental Protocol 

During the six-day experiment, the crayfish were fed commercial aquafeeds once a day. The crayfish were divided into four treatment groups: a control group (saline, Control), an LPS treatment group (1 mg kg^−1^ BW, LPS) [35], a DON treatment group (3 mg kg^−1^ BW, DON) [39], and an LPS + DON treatment group (1 mg kg^−1^ LPS + 3 mg kg^−1^ DON BW, L+D). Each group consisted of four tanks (25 L), with each tank containing 12 crayfish. Each crayfish was injected with 100 μL of saline, LPS, DON, or a combination of LPS and DON solutions. LPS was injected eight hours before the time point of DON injection (day 0) [18], using the method provided by Rodríguez et al. [21]. The injections, consisting of saline, LPS, DON, or a combination of LPS and DON solutions, were administered into the coxes of the third pair of pereiopods of the crayfish using a 100 μL Hamilton syringe.

### 5.3. Survival Rate

During the six-day experiment, the number of crayfish deaths in each group (=12) was recorded daily, and the survival rate was calculated as the number of surviving crayfish divided by the initial number of crayfish [40].

### 5.4. Enzyme Activity Analysis

At 3 and 6 h, four crayfish were randomly chosen from each group, and blood samples were taken from the pericardial cavity of the crayfish. The sera were collected by centrifugation at 3000× *g* for 10 min and stored at −80 °C until analysis [41]. After the sera were thawed on ice, the activities of SOD, CAT, GST, and AKP were measured using commercial kits in accordance with the manufacturer’s instructions (Nanjing Jiancheng Bioengineering Institute, Nanjing, China). Each sample was analyzed in triplicate, and blank and standard controls were included in each analysis to validate the findings and maintain consistency.

### 5.5. RNA Isolation and Quantitative RT-qPCR

At 3 and 6 h, four crayfish were randomly chosen from each group, and hepatopancreas samples were obtained from the crayfish. The samples were washed twice with ice-cold PBS (pH 7.4), and the total RNA was extracted using TriPure Isolation Reagent (Roche, Indianapolis, IN, USA). The RNA quantity was measured using the NanoDrop^TM^ 2000 spectrophotometer (Thermo Fisher Scientific, Wilmington, NC, USA), while the quality was evaluated through formaldehyde agarose gel electrophoresis. The total RNA was reverse transcribed into cDNA, and the housekeeping gene 18S rRNA (GenBank: AF436001) was used as an internal control. The relative gene expression levels were determined using the 2^−ΔΔCT^ method [42]. Real-time PCR was conducted using the LightCycler^®^ 480 Real-Time PCR System (Roche Diagnostics GmbH, Mannheim, Germany). The primer pair sequences are shown in Table 1.

### 5.6. Histopathological Analysis

On days three and six, hepatopancreas samples were collected for histopathological analysis. The samples were excised and fixed in 40 g L^−1^ paraformaldehyde at 4 °C overnight [51]. The samples were then dehydrated in ethanol, cleared in dimethyl benzene, and embedded in paraffin. The paraffin-embedded sections (5 μm) were sliced with a paraffin slicer (Leica, Wetzlar, Germany) and stained with hematoxylin and eosin (H&E). Finally, the sections were observed under a light microscope to identify histological changes (Leica, Wetzlar, Germany) [52].

### 5.7. Statistical Analysis

The survival rate was analyzed using the log-rank test, and the curve was generated using the Kaplan–Meier method. The data are presented as mean ± SEM. Two-way analysis of variance (ANOVA) was used to determine the significant difference in the four treatment groups at different time points. For the same time point, one-way analysis of variance (ANOVA) was performed for the four treatment groups, followed by Tukey’s multiple comparison test. Results with *p*-values less than 0.05 were considered statistically significant. The data were analyzed using GraphPad Prism ver. 501 statistical software program (GraphPad Software, San Diego, CA, USA). 

## Figures and Tables

**Table 1:** Primers of *P. clarkii* used in the experiment.

Name	Sequence (5’-3’)	Reference
Toll sense	GCTGTTGCTGCTTAGGCTCA	[43]
Toll antisense	TCCTCCACAGCTCTTCATTCC	
Cactus sense	CTTGTGAGAGAGCCGTGTG	[43]
Cactus antisense	CAGTACAAGCAGCAGCAGCA	
Spätzle sense	GTCGGCAGCAACGACATACA	[43]
Spätzle antisense	GGTGTCATGGTTGGCTGTGA	
PcBGRPRT sense	CCCACGCTGACTATTCGG	[36]
PcBGRPRT antisense	GGTTGTCCAGGGAGTTGTCG	
Dorsal sense	TCACTGTTGACCCACCTTAC	[44]
Dorsal antisense	GGAAAGGGTCCACTCTAATC	
ALF8 sense	GGGGGAAGCGATGACGAG	[45]
ALF8 antisense	GACGGGTTGGCACAAGAGC	
gst sense	ACTTAGAGACGGACTTCCAG	[46]
gst antisense	CGAGGGCGAACTTCACGG	
PcCru4 sense	CTCTGACTGCCAGGTGTTT	[47]
PcCru4 antisense	TGCGAGCTGTGATGGTTAG	
akp sense	CCACACTACGTGGCAGCAGCGAC	[48]
akp antisense	GCCAGTGAAGAGGTGGGCATGG	
cat sense	GCTGAGGTGGAACAGATGGCA	[49]
cat antisense	AAGGGAATCAGACCGTGAGTGATC	
sod sense	CAAATCAGTGGCAGGCTGGAAA	[49]
sod antisense	CAAATCAGTGGCAGGCTGGAAA	
PcCTSL sense	CGGATCACTGGAGGGTCAAACACTT	[35]
PcCTSL antisense	GCAATTTTCATCCTCGGCATCAT	
18S sense	TCTTCTTAGAGGGATTAGCGG	[50]
18S antisense	AAGGGGATTGAACGGGTTA	

## Data Availability

Not applicable.
